# Genome of the Lord Howe Island Stick Insect Reveals a Highly Conserved Phasmid X Chromosome

**DOI:** 10.1093/gbe/evad104

**Published:** 2023-06-03

**Authors:** Oliver P Stuart, Rohan Cleave, Michael J L Magrath, Alexander S Mikheyev

**Affiliations:** Research School of Biology, Australian National University, Canberra, ACT, Australia; Zoos Victoria, Parkville, Victoria, Australia; Zoos Victoria, Parkville, Victoria, Australia; School of Biosciences, University of Melbourne, Melbourne, Australia; Research School of Biology, Australian National University, Canberra, ACT, Australia

**Keywords:** phasmids, conservation genetics, X chromosome, large genomes

## Abstract

We present a chromosome-scale genome assembly for *Dryococelus australis*, a critically endangered Australian phasmid. The assembly, constructed with Pacific Biosciences continuous long reads and chromatin conformation capture (Omni-C) data, is 3.42 Gb in length with a scaffold N50 of 262.27 Mb and L50 of 5. Over 99% of the assembly is contained in 17 major scaffolds, which corresponds to the species’ karyotype. The assembly contains 96.3% of insect Benchmarking Unique Single Copy Ortholog genes in single copy. A custom repeat library identified 63.29% of the genome covered by repetitive elements; most were not identifiable based on similarity to sequences in existing databases. A total of 33,793 putative protein-coding genes were annotated. Despite the high contiguity and single-copy Benchmarking Unique Single Copy Ortholog content of the assembly, over 1 Gb of the flow-cytometry-estimated genome size is not represented, likely due to the large and repetitive nature of the genome. We identified the X chromosome with a coverage-based analysis and searched for homologs of genes known to be X-linked across the genus *Timema*. We found 59% of these genes on the putative X chromosome, indicating strong conservation of X-chromosomal content across 120 million years of phasmid evolution.

**Significance**

Stick insects are a diverse insect order comprising over 3,000 recorded species, yet only one genus is represented among publicly available chromosome-scale assemblies. This genus, *Timema*, is the outgroup of all other extant stick insects, and so the diversity of the order is severely underrepresented. *Dryococelus australis* is a critically endangered Australian stick insect, currently bred in captivity and with a highly restricted range and population size in the wild. Population genetic studies of the species have been recommended for over a decade now. To this end, we present a chromosome-scale genome assembly for *D. australis*. This assembly is highly complete and assembled into scaffolds corresponding to the species’ karyotype. This assembly significantly expands the genomic resources available for the study of phasmids and will facilitate future population genomic studies of an iconic Australian insect and flagship species for invertebrate conservation.

## Introduction

*Dryococelus australis*, the Lord Howe Island (LHI) stick insect, is a critically endangered (Rudolf and Brock 2017) Australian phasmid. Once found in abundance on LHI in the Tasman Sea, *D. australis* was driven locally extinct following the accidental introduction of black rats (*Rattus rattus*) in 1918. In 2001, a population was discovered on Ball's Pyramid (BP), a sea stack about 20 km southeast of LHI (Priddel et al. 2003), and shortly after, a mating pair was taken from BP and used to found a captive breeding program at the Melbourne Zoo in Melbourne, Australia. Inbreeding depression was detected in the captive population shortly after its founding (Honan 2008) and a genetic study has been recommended by previous conservation managers of the species (Priddel et al. 2003; Honan 2008; Carlile et al. 2009). An initial study recovered complete mitochondrial genomes from extinct LHI and captive specimens, confirming the conspecificity of the BP and LHI populations (Mikheyev et al. 2017). This study also used short read sequence data to assemble a draft genome (GenBank assembly accession GCA_002236955.1), which showed signs of polyploidy but also had very low contiguity (N50 = 17,265). In this study, we present a chromosome-scale, annotated genome assembly for *D. australis* and place the species in a phylogenetic context. We also investigate X chromosome conservation by leveraging existing phasmid genomic data. Presently, the only published chromosome-scale phasmid assembly is of *Timema cristinae* (GCA_002928295.1), a genus that forms the outgroup to all other phasmids and which is estimated to have diverged from *D. australis* 121.8 (95% confidence interval: 105.1–139.4) million years ago (Simon et al. 2019). The assembly presented herein expands the scope of available phasmid genomic resources and will facilitate future population and comparative genomic studies of *D. australis*, a flagship species for invertebrate conservation worldwide.

## Results and Discussion

### Karyotype

**Fig. 1.**
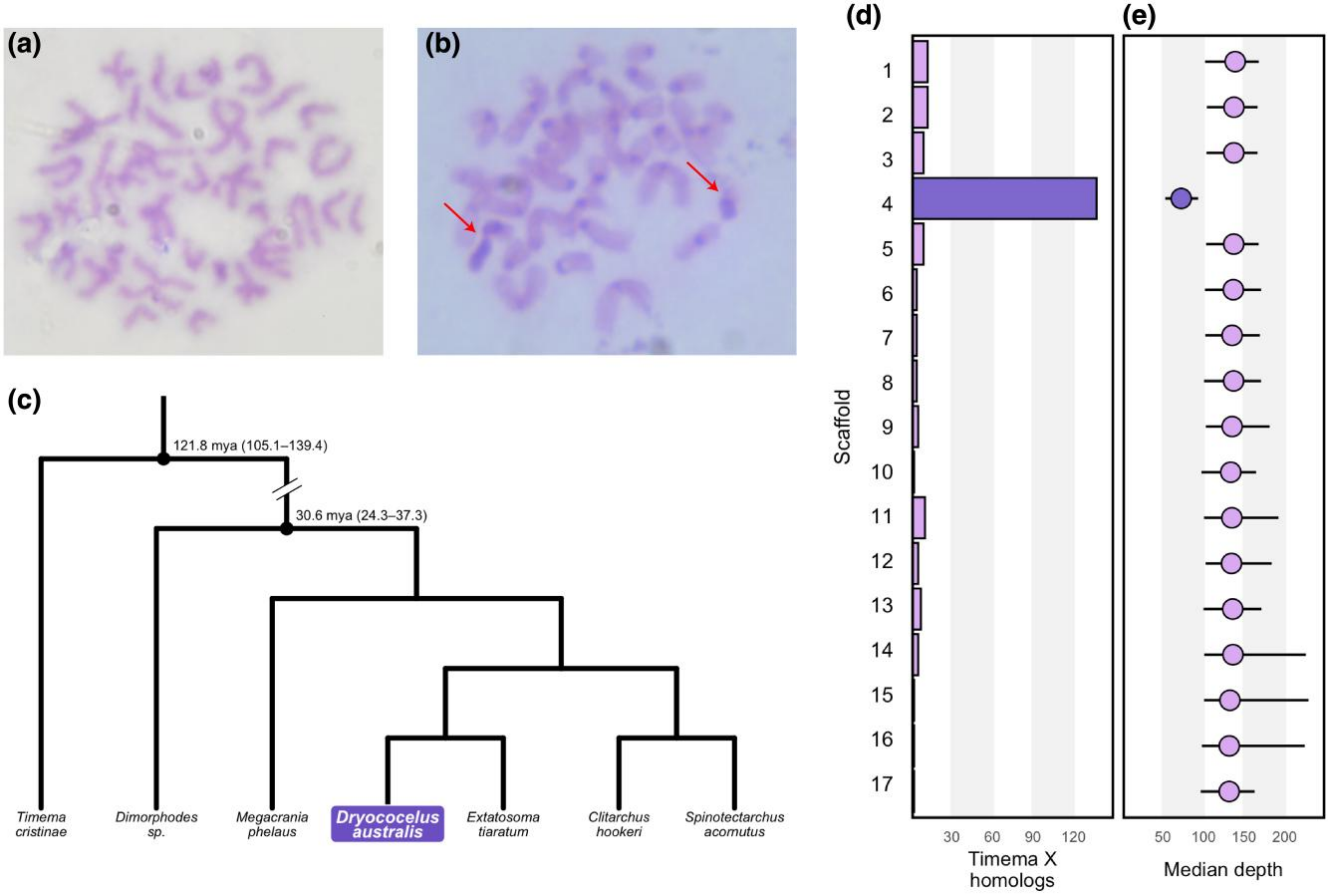
Representative metaphase plates of *Dryococelus australis* embryonic tissue taken before (*a*) and after (*b*) digestion in saturated BaOH. The arrows indicate highly heterochromatic heteromorphic bodies, which we infer to be the sex chromosomes (X and Y). All slides were stained with a 5% Giemsa solution and observed under 1,000× with a compound light microscope and photographed with a mounted Nikon D90. (*c*) Subtree showing the topology of the maximum likelihood tree constructed from 774 amino acid alignments using transcriptome data from Simon et al. (2019) and this study. Only the Lanceocercata, the antipodean stick insects, and *Timema cristinae* are shown. Divergence times are taken from Simon et al. (2019). (*d*) Best BLAST hit location of X-linked genes in *Timema* from Parker et al. (2022). (*e*) Median GC normalized read depth of coverage of PacBio CLR aligned to the major scaffolds of the final assembly. The error bars show 95th percentile ranges of mean depth in 10 kb bins.

Direct observation of chromosomes identified a diploid karyotype of 2N = 34 (fig. 1*a*). C-banding demonstrated mostly uninformative heterochromatin distributions with only the centromeres differentiated except for two chromosomes per individual that differentially stained across their entire length (fig. 1*b*). We infer these to be the sex chromosomes. The sex of the embryos dissected was unknown but the individual depicted in figure 1*b* has one large and one small putative sex chromosome, implying XY sex determination which has been observed in other stick insects (Maniko 1951).

### Assembly Evaluation

Initial assembly of 31,640,841 reads (N50 = 35,056 after removing reads ≤20 kb) with WTDBG2 followed by removal of potential contaminants and secondary haplotypes generated an assembly of 2,788 contigs with N50 and L50 values of 13.48 Mb and 76 (table 1). Mapping of 486,619,719 OmniC read pairs to this intermediate assembly and scaffolding by HiRise joined 905 contigs and split 3, resulting in a final assembly of 1,879 scaffolds with scaffold N50 and L50 values of 262.28 Mb and 5. This is an almost 30,000-fold increase in N50 compared with the original short read assembly (table 1). At 3.42 Gb, the scaffold assembly is 30 Mb shorter than the short read assembly (table 1), suggesting the presence of duplicate contigs and contamination in the latter. Seventeen large (>60 Mb) scaffolds comprise 99.4% of the length of this assembly, corresponding to the observed karyotype of 2N = 34 (fig. 1*a* and *b*). We mapped the OmniC reads back to these scaffolds and plotted the resultant contact matrix (supplementary fig. S1, Supplementary Material online). “Scaffold_4” shows fewer contacts with other scaffolds, linking it to the two highly heterochromatic chromosomes identified with C-banding (fig. 1*b*). We assessed completeness of the scaffold assembly by searching for Benchmarking Unique Single Copy Ortholog (BUSCO) genes from the insecta_odb10 database. The genome contains complete single-copy sequences of 96.3% of insect BUSCOs with 0.7% present but duplicated (table 1). Only 46.7% were identifiable as single copies in the original short-read assembly (table 1).

**Table 1:** Summary Statistics of *Dryococelus australis* Genome Assemblies

Publication	Mikheyev et al. (2017)	This study
Contiguity		
# Contigs	357,088	2,788
Contig N50 (bp)	17,527	13,476,729
Contig L50	56,852	76
# Scaffolds	NA	1,879
Scaffold N50 (bp)	NA	262,277,254
Scaffold L50	NA	5
Length (bp)	3,451,380,184	3,421,391,311
GC content (%)	38.6	38.5
Completeness: genome (BUSCO v5.4.4; insecta_odb10, *n* = 1,367)		
Complete single copy	639 (46.7%)	1,316 (96.3%)
Complete duplicated	27 (2.0%)	10 (0.7%)
Fragmented	509 (37.2%)	12 (0.9%)
Missing	192 (14.1%)	29 (2.1%)
Completeness: predicted proteins (BUSCO v5.4.4; insecta_odb10, *n* = 1,367)		
Complete single copy	NA	706 (51.4%)
Complete duplicated	NA	6 (0.4%)
Fragmented	NA	111 (8.1%)
Missing	NA	548 (40.1%)

### Annotation

After mapping of our RNA-seq data to the completed genome assembly with STAR, we found only 88.41% of all read pairs mapped successfully. A combination of de novo and homology-based approaches identified 37,538 predicted protein-coding genes, 3,745 of which were flagged as errors by NCBI's automated submission portal due to internal stop codons, leaving 33,793 predicted protein-coding genes and 682 predicted tRNAs. Of these predicted protein-coding genes, 6,932 could be assigned putative homologs. Previous phasmid assemblies have reported similar or greater numbers of predicted protein-coding genes (*Clitarchus hookeri*, *n* = 66,470 [Wu et al. 2017]; *Medauroidea extradentata*, *n* = 35,742 [Brand et al. 2018]); however, *Timema* annotations tend to have 11,000 to 16,000 (Jaron et al. 2022; BioProject accession PRJEB31411). We also ran BUSCO on our predicted protein sequences after taking the longest isoform per gene and found only 51.4% and 53.8% of insect BUSCO genes in single copy in the protein sequences filtered and unfiltered by the NCBI submission portal respectively (table 1; only NCBI filtered proteins reported). This is lower than for the official gene set published with the *C. hookeri* assembly which contained 73.1% of arthropod BUSCO genes (Wu et al. 2017) although their single copy or duplicate status was not reported. Our protein annotation is therefore a poor representation of the total gene content of the *D. australis* genome, even though we used multiple adult tissues from both sexes. This is likely explained by our input data. Assembly of PacBio CLR reads produces contigs with many indel errors which can drastically impact the accuracy of protein prediction (Watson and Warr 2019). As a result, the set of protein sequences presented here only represents a fraction of the true CDS sequences present in the assembly. This does not affect the BUSCO results for the whole-genome sequence, as BUSCO detects intron–exon boundaries *de novo* in this mode and is likely more tolerant of sequencing error than our annotation approach. This shortfall in our predicted proteins might be alleviated by short read polishing to correct indel errors introduced by the long error-prone PacBio CLR reads. The assembly was highly repetitive. After filtering, 2.17 Gb (63.29%) of the assembly was masked by RepeatMasker (supplementary table S1, Supplementary Material online). The majority of these, 1.56 Gb, were not classifiable based on sequences in existing databases (supplementary table S1, Supplementary Material online). The most abundant elements were DNA transposons (9.45%) and long terminal repeats (5.18%; supplementary table S1, Supplementary Material online), and there was a clear positive relationship between scaffold length and repetitive element composition overall, although SINEs show the opposite trend (supplementary fig. S2, Supplementary Material online). Kimura 2 parameter divergence landscapes imply ongoing proliferation of rolling circle and satellite elements as well as a recent concurrent burst of DNA, LINE, and SINE elements (supplementary fig. S3, Supplementary Material online).

### Genome Size

Other assemblies of large polyneopteran genomes contain high numbers of recently diverged transposable and interspersed repetitive elements (Wang et al. 2014; Wu et al. 2017; Verlinden et al. 2021) and the positive relationship between eukaryotic genome size and the total number/cumulative sequence length of transposable elements is well established (Kidwell 2002; Lee and Kim 2014). High transposable and repetitive element load increases the chances of ectopic recombination and thus comes at a fitness cost. This creates an evolutionary arms race between transposable element expansion and the host's own evolved containment mechanisms (reviewed in Kelleher et al. 2020). The extant *D. australis* population (from which the assembly presented herein was derived) has experienced a recent colonization bottleneck and subsequent low population size (Carlile et al. 2009). In *Arabidopsis lyrata*, bottlenecked populations show increased transposable element (TE) transposition (Lockton et al. 2008), consistent with natural selection acting to suppress their proliferation. We suggest that genome size in *D. australis* has expanded partly due to the release of TEs from host containment as a result of the attenuated power of selection in this small, bottlenecked wild population. We previously estimated the size of the *D. australis* genome with flow cytometry at roughly 4.7 Gb so despite the high contiguity of the assembly, a large percentage (roughly 27.7%) of the true genome sequence remains unassembled. Recent bursts of transposable and repetitive elements, which we believe is likely and is borne out by the divergence landscapes of the major repeat classes (supplementary fig. S3, Supplementary Material online), would create the conditions required for this shortfall in the assembly: highly similar interspersed and tandemly duplicated sequences that cannot be spanned by our long reads. The N50 of the PacBio reads used in the contig assembly was 35,056 and the longest detected repeat region masked by RepeatMasker was 42,143. We believe it is likely that many more long repeat sequences remain undetected due to collapse in the assembly. The annotated protein-coding genes had a mean intron length per transcript of 1,424 (supplementary table S1, Supplementary Material online). These introns are considerably smaller than expected based on previously estimated relationships between genome size and intron size (Wang et al. 2014, Supplementary Figure S9). In fact, the mean intron size in the *D. australis* genome assembly is not much larger than that of the *Drosophila melanogaster* assembly presented by (Adams et al. 2000; Verlinden et al. 2021). We believe this further supports our assertion that a large proportion of the *D. australis* genome sequence remains inaccessible with the sequencing technology we have employed. Indeed, even ultra-long reads generated by Oxford Nanopore technology could only assembly 85.8% of the full length of the human genome (Jain et al. 2018), which is considerably less burdened by repetitive and transposable elements than that of *D. australis*.

### Phylogenetics

We generated a concatenated supermatrix of 164,692 amino acid residues across 774 orthologs for Phasmatodea. Mean missingness across taxa was 3.96% (1.10–10.55); *D. australis* had 2.21% characters missing (supplementary table S2, Supplementary Material online). With maximum likelihood inference, we obtained an almost identical topology to Simon et al. (2019) except for the placement of *Bacillus rossius* and *Phyllium philippinicum*, the branching of which had low bootstrap support. Here, we present only the subtree around our focal *D. australis*. We find that *Extatosoma tiaratum*, an east Australian endemic, is the closest sampled relative of *D. australis* (fig. 1*c*). This places *D. australis* within the Australian Lanceocercata, corroborating previous findings based on mitochondrial genomes (Forni et al. 2021) and Sanger sequencing data sets (Buckley et al. 2009, 2010) that placed *D. australis* into a clade with *Eurycnema* spp. The latter studies inferred an Australian ancestral range and estimated the age of the *D. australis* lineage as much older than LHI, suggesting dispersal from Australia across now submerged islands.

### X Chromosome Identification

We used tblastn to identify putative homologs of X-linked genes which are conserved across five *Timema* species (Parker et al. 2022). The majority (59%) of the best hits were found on Scaffold_4 (fig. 1*d*), and the remainder mapped to the other major scaffolds mostly in proportion to their length, for example, the next scaffold with the most hits was Scaffold_1, the longest scaffold. Scaffold_11 is a notable outlier to this trend (fig. 1*d*), harboring 10 best hits compared with Scaffold_3 which harbored 9, despite being almost three times as long. We also remapped the PacBio reads generated from the male sample used in the initial assembly back to the major scaffolds and calculated GC normalized depth in 10 kb bins. This same scaffold had roughly 50% mean depth of coverage compared with the other scaffolds (fig. 1*e*). Finally, Scaffold_4 is also an outlier in repetitive element composition, overall and for specific element types (supplementary fig. S2, Supplementary Material online). This strongly implicates Scaffold_4 as the X chromosome. Parker et al. (2022) found high conservation of these *Timema* X genes on the *B. rossius* X chromosome; this study extends this finding more broadly across Phasmatodea. Repeated observation of heteromorphy in C-banded *D. australis* embryos (fig. 1*b*) suggests the presence of a Y chromosome not apparent in the assembly. We attempted polymerase chain reaction (PCR) primer design from sequences of minor scaffolds showing inflated coverage in male versus female samples (with sequencing reads from another ongoing project) but no primers showed successful differential amplification from male and female gDNA extracts, and so we are unable to confirm the true sex determination system of *D. australis*.

## Materials and Methods

### Karyotype

We used Giemsa staining with BaOH C-banding (Ghosh and Singh 1975) to observe the karyotype of *D. australis*. Eggs from the captive colony were dissected at 2–3 months after laying and the embryos placed in distilled water for 10 min, washed twice in a 3:1 solution of methanol and glacial acetic acid, and finally incubated in this same solution for 2 h at room temperature. Embryos were then macerated on a glass microscope slide in a 3:2 solution of glacial acetic acid and distilled water using a blunt brass rod. Extraneous material was removed with forceps and the cellular suspension rolled across the surface of the slide. The slide was dried on a hotplate set to 35 °C for 15 min. Slides were then incubated in saturated BaOH solution for 5 min followed by washes in distilled water, 0.2 M HCl, distilled water again, and a final 30 min incubation in 2X saline sodium citrate at room temperature. Slides were washed with a solution of 1% w/v KH_2_PO_4_ 2% w/v Na_2_HPO_4_12H_2_O and stained in a 5% v/v solution of Giemsa in the same solution. Slides were visualized at 1,000× with a compound light microscope and imaged with a mounted Nikon D90.

### Reference Genome Assembly

We sampled a single adult male *D. australis* from the Melbourne Zoo captive population for the assembly. The reference genome sequence was assembled and annotated by Dovetail Genomics; we briefly describe the method here. High-molecular weight DNA was extracted from snap frozen muscle tissue. About 588.2 Gb of PacBio CLR reads were generated with this extract. Contigs were assembled with WTDBG v2.5 (Ruan and Li 2020) with an estimated genome size of 4.7 Gb (based on flow cytometric estimates, data not shown), minimum read length of 20,000, and a minimum alignment length of 8,192. Contaminant contigs were identified with blobtools v1.1.1 (Laetsch and Blaxter 2017) and removed. Secondary haplotypes were removed with purge_dups v1.2.3 (Guan et al. 2020). Proximity ligation information was generated with 150 bp Omni-C libraries. Tissue from the same individual was fixed in formaldehyde and then DNA was extracted, followed by digestion with DNAse I, end repair, ligation to a biotinylated bridge adapter, and finally adapter ligation. After ligation, crosslinks were reversed and the DNA was purified. Purified DNA was treated to remove biotin not internal to ligated fragments. Sequencing libraries were generated using NEBNext Ultra enzymes (New England Biolabs). Biotin-containing fragments were isolated using streptavidin beads before PCR enrichment of each library. The library was sequenced on an Illumina HiSeqX platform to produce approximately 30× sequence coverage. Omni-C reads were mapped to the contig assembly with bwa mem (Li 2013), and HiRise (Putnam et al. 2016) was used to order and orient contigs into scaffolds. We assessed completeness of the scaffold assembly using BUSCO analysis software v5.4.4 (Manni et al. 2021) with the BUSCO database insecta_odb10 containing 1,367 protein sequences.

### Annotation

Repeat families were identified *de novo* and classified using the software package RepeatModeler v2.0.1 (Smit et al. 2021). We further filtered repeats by a BLAST search to the “nr” database and removed any unclassified family whose best hit was a known protein not originating from a transposable element, including mitochondrial proteins. We then used RepeatMasker v2.0.1 (Smit et al. 2021) to calculate the divergence among repeat families without CpG correction and plotted the repeat landscape in R v4.1.2 (R Core Team 2018) with ggplot2 (Wickham 2016). Coding sequences from *C. hookeri* (Wu et al. 2017), *M. extradentata* (Brand et al. 2018), and *T. cristinae* (Soria-Carrasco et al. 2014) were used to train the ab initio model for *D. australis* using both AUGUSTUS software v2.5.5 (Stanke et al. 2008) and SNAP version 2006-07-28 (Korf 2004). We extracted RNA from tissue samples of both sexes of the ventral ganglion, leg muscle, gut lumen, Malpighian tubules, and gonads with RNEasy spin column kits (Qiagen); libraries were prepared and sequenced at BGI Hong Kong on a BGISEQ-500 instrument in paired end mode with 150 bp reads. Reads were mapped onto the assembly using STAR v2.7 (Dobin et al. 2013) and intron hints generated with the bam2hints tools within the AUGUSTUS software. SNAP and AUGUSTUS (with intron–exon boundary hints provided from RNA-Seq) were then used to predict genes in the repeat masked assembly. Only gene models that were predicted by both SNAP and AUGUSTUS were retained. Genes were further characterized for their putative function by performing a BLAST search of the peptide sequences against a set of protein sequences from UniProt. Finally, to check the completeness of our annotation, we ran BUSCO in protein mode with the nsecta_odb10 database on our final set of predicted protein sequences.

### Phylogenetic Analysis

We placed *D. australis* into a recently published phasmid phylogeny (Simon et al. 2019), using a *de novo* assembled transcriptome. We assembled all RNA-Seq reads generated for annotation with Trinity (Henschel et al. 2012) and used all phasmid data from Simon et al. (2019). We clustered the sequences of each species with CD-HIT-EST v4.8.1 (Li and Godzik 2006) with a 95% sequence identity threshold. We constructed an ortholog database with Orthograph v0.7.1 (Petersen et al. 2017) using the OrthoDB official gene sets for *Ephemera danica*, *Ladona fulva*, *Rhodnius prolixus*, and *Zootermopsis nevadensis* (Zdobnov et al. 2021) and identified orthologs in the phasmid transcriptomes. We aligned resultant amino acid sequences with MAFFT v7.508 (Katoh and Standley 2013), using the default settings and removed poorly aligned bases with TrimAl v1.4.1 (Capella-Gutiérrez et al. 2009) in “automated1” mode. For each alignment, we removed taxa that had >50% missing characters compared with the maximum sequence length. Finally, we selected only alignments that had >80% representation of the full taxon list, always including *D. australis*. We constructed NJ trees of all alignments in R with apetools v5.5 (Paradis and Schliep 2019) and visually inspected alignments, manually removed any taxa with obviously outlying branch lengths using a bash script. We removed paralog alignments by identifying trees whose length was dominated by long internal branches that split taxa into >1 clades not corresponding to the published phylogeny (Simon et al. 2019). We filtered for missingness again as above. Finally, we constructed a single nexus file, concatenating all 774 alignments that passed filtering and retaining partitions corresponding to the original clusters of orthologous groups and estimated a maximum likelihood tree using IQ-TREE v1.6.12 (Nguyen et al. 2015; Minh et al. 2020). We used PartitionFinder (Lanfear et al. 2012) and ModelFinder (Chernomor et al. 2016; Kalyaanamoorthy et al. 2017) to find the best supported partitions and substitution models. We calculated branch support with 1,000 ultrafast bootstraps (Hoang et al. 2018).

### X Chromosome Identification

We remapped the long reads used in the original assembly back to the final assembly with minimap v2.21 (Li 2018) and estimated the depth across the major scaffolds in 10 kb bins with mosdepth v0.3.1 (Pedersen and Quinlan 2018) and normalized this by GC content. We then calculated the median and 95% percentile range of depth across all bins for each scaffold. We obtained amino sequences of genes conserved across five *Timema* species (Parker et al. 2022) and used tBLASTn v2.11.0 (Madden 2013) to identify putative homologs in our assembly, retaining the best hit per input sequence based on *e*-values.

## Supplementary Material

evad104_Supplementary_Data

## Data Availability

All raw data, including fastq files for Omni-C, RNA, and PacBio continuous long-read sequencing, as well the assembly and annotation in fasta and gff formats, respectively, have been submitted to the National Centre for Biotechnology Information databases and are available under BioProject PRJNA930028. The authors also provide additional files in an Open Science Framework repository (https://osf.io/9v487/), including the original annotation gff file prior to filtering using NCBI's automated submission portal, as well as the fasta library of identified repeat families and a gff file of repetitive features.

## References

[evad104-B1] Adams MD , et al 2000. The genome sequence of *Drosophila melanogaster*. Science 287:2185–2195. 10731132 10.1126/science.287.5461.2185

[evad104-B2] Brand P , Lin W, Johnson BR. 2018. The draft genome of the invasive walking stick, *Medauroidea extradendata*, reveals extensive lineage-specific gene family expansions of cell wall degrading enzymes in phasmatodea. G3 (Bethesda). 8:1403–1408. 29588379 10.1534/g3.118.200204 PMC5940134

[evad104-B3] Buckley TR , Attanayake D, Bradler S. 2009. Extreme convergence in stick insect evolution: phylogenetic placement of the Lord Howe Island tree lobster. Proc R Soc B: Biol Sci. 276:1055–1062. 10.1098/rspb.2008.1552 PMC2679072 19129110

[evad104-B4] Buckley TR , Attanayake D, Nylander JA, Bradler S. 2010. The phylogenetic placement and biogeographical origins of the New Zealand stick insects (Phasmatodea). Syst Entomol. 35:207–225.

[evad104-B5] Capella-Gutiérrez S , Silla-Martínez JM, Gabaldón T. 2009. trimAl: a tool for automated alignment trimming in large-scale phylogenetic analyses. Bioinformatics 25:1972–1973. 19505945 10.1093/bioinformatics/btp348 PMC2712344

[evad104-B6] Carlile N , Priddel D, Honan P. 2009. The recovery programme for the Lord Howe Island Phasmid (*Dryococelus australis*) following its rediscovery. Ecol Manag Restor. 10:S124–S128.

[evad104-B7] Chernomor O , von Haeseler A, Minh BQ. 2016. Terrace aware data structure for phylogenomic inference from supermatrices. Syst Biol. 65:997–1008. 27121966 10.1093/sysbio/syw037 PMC5066062

[evad104-B8] Dobin A , et al 2013. STAR: ultrafast universal RNA-seq aligner. Bioinformatics 29:15–21. 23104886 10.1093/bioinformatics/bts635 PMC3530905

[evad104-B9] Forni G , et al 2021. Phylomitogenomics provides new perspectives on the Euphasmatodea radiation (Insecta: Phasmatodea). Mol Phylogenet Evol. 155:106983. 33059069 10.1016/j.ympev.2020.106983

[evad104-B10] Ghosh P , Singh IP. 1975. Modified method of c banding using barium hydroxide. Acta Genet Med Gemellol (Roma). 24:315–316. 68655 10.1017/s0001566000010448

[evad104-B11] Guan D , et al 2020. Identifying and removing haplotypic duplication in primary genome assemblies. Bioinformatics 36:2896–2898. 31971576 10.1093/bioinformatics/btaa025 PMC7203741

[evad104-B12] Henschel R et al 2012. Trinity RNA-Seq assembler performance optimization. In: Proceedings of the 1st Conference of the Extreme Science and Engineering Discovery Environment: Bridging from the eXtreme to the Campus and Beyond; 2012 Jul 16–20; Chicago, IL. New York (NY): Association for Computing Machinery. p. 45.

[evad104-B13] Hoang DT , Chernomor O, von Haeseler A, Minh BQ, Vinh LS. 2018. UFBoot2: improving the ultrafast bootstrap approximation. Mol Biol Evol. 35:518–522. 29077904 10.1093/molbev/msx281 PMC5850222

[evad104-B14] Honan P . 2008. Notes on the biology, captive management and conservation status of the Lord Howe Island Stick Insect (*Dryococelus australis*) (Phasmatodea). J Insect Conserv. 12:399–413.

[evad104-B15] Jain M , et al 2018. Nanopore sequencing and assembly of a human genome with ultra-long reads. Nat Biotechnol. 36:338–345. 29431738 10.1038/nbt.4060 PMC5889714

[evad104-B16] Jaron KS , et al 2022. Convergent consequences of parthenogenesis on stick insect genomes. Sci Adv. 8:eabg3842. 35196080 10.1126/sciadv.abg3842 PMC8865771

[evad104-B17] Kalyaanamoorthy S , Minh BQ, Wong TKF, von Haeseler A, Jermiin LS. 2017. ModelFinder: fast model selection for accurate phylogenetic estimates. Nat Methods. 14:587–589. 28481363 10.1038/nmeth.4285 PMC5453245

[evad104-B18] Katoh K , Standley DM. 2013. MAFFT multiple sequence alignment software version 7: improvements in performance and usability. Mol Biol Evol. 30:772–780. 23329690 10.1093/molbev/mst010 PMC3603318

[evad104-B19] Kelleher ES , Barbash DA, Blumenstiel JP. 2020. Taming the turmoil within: new insights on the containment of transposable elements. Trends Genet. 36:474–489. 32473745 10.1016/j.tig.2020.04.007 PMC7347376

[evad104-B20] Kidwell MG . 2002. Transposable elements and the evolution of genome size in eukaryotes. Genetica 115:49–63. 12188048 10.1023/a:1016072014259

[evad104-B21] Korf I . 2004. Gene finding in novel genomes. BMC Bioinformatics. 5:59. 15144565 10.1186/1471-2105-5-59 PMC421630

[evad104-B22] Laetsch DR , Blaxter ML. 2017. BlobTools: interrogation of genome assemblies. F1000Research 6:1287.

[evad104-B23] Lanfear R , Calcott B, Ho SYW, Guindon S. 2012. PartitionFinder: combined selection of partitioning schemes and substitution models for phylogenetic analyses. Mol Biol Evol. 29:1695–1701. 22319168 10.1093/molbev/mss020

[evad104-B24] Lee S-I , Kim N-S. 2014. Transposable elements and genome size variations in plants. Genomics Inform. 12:87–97. 25317107 10.5808/GI.2014.12.3.87 PMC4196380

[evad104-B25] Li H. 2013. Aligning sequence reads, clone sequences and assembly contigs with BWA-MEM. doi:10.48550/arXiv.1303.3997.

[evad104-B26] Li H . 2018. Minimap2: pairwise alignment for nucleotide sequences. Bioinformatics 34:3094–3100. 29750242 10.1093/bioinformatics/bty191 PMC6137996

[evad104-B27] Li W , Godzik A. 2006. Cd-hit: a fast program for clustering and comparing large sets of protein or nucleotide sequences. Bioinformatics 22:1658–1659. 16731699 10.1093/bioinformatics/btl158

[evad104-B28] Lockton S , Ross-Ibarra J, Gaut BS. 2008. Demography and weak selection drive patterns of transposable element diversity in natural populations of *Arabidopsis lyrata*. Proc Natl Acad Sci USA. 105:13965–13970. 18772373 10.1073/pnas.0804671105 PMC2544562

[evad104-B29] Madden T . 2013. The BLAST sequence analysis tool. In: The NCBI handbook. Vol. 2. Bethesda (MD): National Center for Biotechnology Information. p. 425–436.

[evad104-B30] Maniko S . 1951. An atlas of the chromosome numbers in animals. 2nd ed. Ames (IA): Iowa State College Press.

[evad104-B31] Manni M , Berkeley MR, Seppey M, Simão FA, Zdobnov EM. 2021. BUSCO update: novel and streamlined workflows along with broader and deeper phylogenetic coverage for scoring of eukaryotic, prokaryotic, and viral genomes. Mol Biol Evol. 38:4647–4654. 34320186 10.1093/molbev/msab199 PMC8476166

[evad104-B32] Mikheyev AS , et al 2017. Museum genomics confirms that the Lord Howe Island stick insect survived extinction. Curr Biol. 27:3157–3161. 28988864 10.1016/j.cub.2017.08.058

[evad104-B33] Minh BQ , et al 2020. IQ-TREE 2: new models and efficient methods for phylogenetic inference in the genomic era. Mol Biol Evol. 37:1530–1534. 32011700 10.1093/molbev/msaa015 PMC7182206

[evad104-B34] Nguyen L-T , Schmidt HA, von Haeseler A, Minh BQ. 2015. IQ-TREE: a fast and effective stochastic algorithm for estimating maximum-likelihood phylogenies. Mol Biol Evol. 32:268–274. 25371430 10.1093/molbev/msu300 PMC4271533

[evad104-B35] Paradis E , Schliep K. 2019. ape 5.0: an environment for modern phylogenetics and evolutionary analyses in R. Bioinformatics 35:526–528. 30016406 10.1093/bioinformatics/bty633

[evad104-B36] Parker DJ , Jaron KS, Dumas Z, Robinson-Rechavi M, Schwander T. 2022. X chromosomes show relaxed selection and complete somatic dosage compensation across Timema stick insect species. J Evol Biol. 35:1734–1750 35933721 10.1111/jeb.14075 PMC10087215

[evad104-B37] Pedersen BS , Quinlan AR. 2018. Mosdepth: quick coverage calculation for genomes and exomes. Bioinformatics 34:867–868. 29096012 10.1093/bioinformatics/btx699 PMC6030888

[evad104-B38] Petersen M , et al 2017. Orthograph: a versatile tool for mapping coding nucleotide sequences to clusters of orthologous genes. BMC Bioinformatics. 18:1–10. 28209129 10.1186/s12859-017-1529-8 PMC5312442

[evad104-B39] Priddel D , Carlile N, Humphrey M, Fellenberg S, Hiscox D. 2003. Rediscovery of the ‘extinct’ Lord Howe Island stick-insect (*Dryococelus australis* (Montrouzier)) (Phasmatodea) and recommendations for its conservation. Biodivers Conserv. 12:1391–1403.

[evad104-B40] Putnam NH , et al 2016. Chromosome-scale shotgun assembly using an in vitro method for long-range linkage. Genome Res. 26:342–350. 26848124 10.1101/gr.193474.115 PMC4772016

[evad104-B41] R Core Team . 2018. R: a language and environment for statistical computing. Vienna (Austria): R Foundation for Statistical Computing. Available from: https://www.R-project.org/.

[evad104-B42] Ruan J , Li H. 2020. Fast and accurate long-read assembly with wtdbg2. Nat Methods. 17:155–158. 31819265 10.1038/s41592-019-0669-3 PMC7004874

[evad104-B43] Rudolf E , Brock P. 2017. *Dryococelus* australis. The IUCN Red List of Threatened Species. [cited 2019 Apr 1]. Available from: 10.2305/IUCN.UK.2017-3.RLTS.T6852A21426226.en.

[evad104-B44] Simon S , et al 2019. Old world and new world phasmatodea: phylogenomics resolve the evolutionary history of stick and leaf insects. Front Ecol Evol. 7:345.

[evad104-B45] Smit A , Hubley R, Green P. 2021. RepeatMasker Open-4.0. Available from: http://repeatmasker.org/.

[evad104-B46] Soria-Carrasco V , et al 2014. Stick insect genomes reveal natural selection's role in parallel speciation. Science 344:738–742. 24833390 10.1126/science.1252136

[evad104-B47] Stanke M , Diekhans M, Baertsch R, Haussler D. 2008. Using native and syntenically mapped cDNA alignments to improve de novo gene finding. Bioinformatics 24:637–644. 18218656 10.1093/bioinformatics/btn013

[evad104-B48] Verlinden H , et al 2021. First draft genome assembly of the desert locust, *Schistocerca gregaria*. F1000Res. 9:775. 10.12688/f1000research.25148.1 PMC7607483 33163158

[evad104-B49] Wang X , et al 2014. The locust genome provides insight into swarm formation and long-distance flight. Nat Commun. 5:2957. doi:10.1038/ncomms3957. 24423660 PMC3896762

[evad104-B50] Watson M , Warr A. 2019. Errors in long-read assemblies can critically affect protein prediction. Nat Biotechnol. 37:124–126. 30670796 10.1038/s41587-018-0004-z

[evad104-B51] Wickham H. 2016. ggplot2: elegant graphics for data analysis. New York: Springer-Verlag. Available from: https://ggplot2.tidyverse.org.

[evad104-B52] Wu C , Twort VG, Crowhurst RN, Newcomb RD, Buckley TR. 2017. Assembling large genomes: analysis of the stick insect (*Cclitarchus hookeri*) genome reveals a high repeat content and sex-biased genes associated with reproduction. BMC Genomics. 18:884. 29145825 10.1186/s12864-017-4245-x PMC5691397

[evad104-B53] Zdobnov EM , et al 2021. OrthoDB in 2020: evolutionary and functional annotations of orthologs. Nucleic Acids Res. 49:D389–D393. 33196836 10.1093/nar/gkaa1009 PMC7779051

