# Supplementary material for: Genome of the Lord Howe Island Stick Insect Reveals a Highly Conserved Phasmid X Chromosome
Source: Genome Biol Evol. 2023 Jun 3;15(6):evad104. doi: 10.1093/gbe/evad104 (PMC10268593; doi:10.1093/gbe/evad104)
Supplement: evad104_Supplementary_Data [file evad104_supplementary_data.docx]

# Supplementary material: Genome of the Lord Howe Island stick insect reveals a highly conserved Phasmid X chromosome

### Oliver P. Stuart^1^*, Rohan Cleave^2^, Michael J.L Magrath^2,3^, Sasha Mikheyev^1^

### (1) Research School of Biology, Australian National University, Canberra ACT 0200, Australia

### (2) Zoos Victoria, Parkville VIC 3052, Australia

### (3) School of Biosciences, University of Melbourne, VIC 3010

*Author for correspondence: Oliver Patrick Stuart, Research School of Biology, Australian National University, Canberra, Australia, oliver.stuart93@gmail.com

Supplementary Table 1. Summary of annotation statistics. Protein annotations were filtered for errors by NCBI’s automated submission portal. Repeats were identified by RepeatMasker v4.1.0 (Smit 2021) and filtered by removing families with low e value BLAST hits to proteins and transcripts from NCBI’s `nr` database.

| Proteins | |
| --- | --- |
| Predicted protein coding genes | 33,793 |
| Mean intron length per transcript (bp) (± SD) | 1,424 (1,792) |
| Mean exon length per transcript (bp) (± SD) | 261 (362) |
| Mean exon number per transcript | 5.80 |
| Predicted tRNAs | 682 |
| Repeats | |
| DNA (bp) (%) | 323,404,957 (9.45) |
| LINE (bp) (%) | 60,411,518 (1.77) |
| SINE (bp) (%) | 46,454,657 (1.36) |
| LTR (bp) (%) | 177,351,614 (5.18) |
| Rolling Circles (bp) (%) | 7,995,429 (0.23) |
| Unknown (bp) (%) | 1,557,661,113 (45.53) |
| Total (bp) (%) | 2,165,283,859 (63.52) |

Supplementary Table 2. List of taxa from Simon et al. (2019) included in phylogenetic tree reconstruction and supermatrix missingness.

| Name | Sites Missing (%) |
| --- | --- |
| *Abrosoma johorensis* | 11329 (6.88) |
| *Achrioptera manga* | 2407 (1.46) |
| *Agamemnon cornutus* | 6408 (3.89) |
| *Agathemera crassa* | 3808 (2.31) |
| *Antongilia madagassa* | 17374 (10.55) |
| *Aretaon asperrimus* | 2083 (1.26) |
| *Bacillus rossius* | 3910 (2.37) |
| *Carausius morosus* | 9329 (5.66) |
| *Clitarchus hookeri* | 9014 (5.47) |
| *Creoxylus spinosus* | 9273 (5.63) |
| *Diapherodes gigantea* | 4453 (2.70) |
| *Dimorphodes* sp. | 3821 (2.32) |
| *Dryococelus australis* | 3634 (2.21) |
| *Epidares nolimetangere* | 5142 (3.12) |
| *Eurycantha calcarata* | 1804 (1.10) |
| *Extatosoma tiaratum* | 3712 (2.25) |
| *Heteropteryx dilatata* | 4662 (2.83) |
| *Libethra strigiventris* | 4361 (2.65) |
| *Medauroidea extradentata* | 4015 (2.44) |
| *Megacrania phelaus* | 2181 (1.32) |
| *Metriophasma diocles* | 5224 (3.17) |
| *Neohirasea fruhstorferi* | 7679 (4.66) |
| *Oreophoetes peruana* | 2504 (1.52) |
| *Orestes mouhotii* | 5006 (3.04) |
| *Orthomeria* sp. | 5319 (3.23) |
| *Orxines xiphias* | 2632 (1.60) |
| *Paranisacantha* sp. | 14679 (8.91) |
| *Peruphasma schultei* | 2591 (1.57) |
| *Phyllium philippinicum* | 4154 (2.52) |
| *Pseudophasma velutinum* | 6617 (4.02) |
| *Pseudosermyle phalangiphora* | 9356 (5.68) |
| *Ramulus artemis* | 13732 (8.34) |
| *Sipyloidea biplagiata* | 12908 (7.84) |
| *Spathomorpha lancettifer* | 13614 (8.27) |
| *Spinotectarchus acornutus* | 12560 (7.63) |
| *Timema cristinae* | 7061 (4.29) |
| *Tirachoidea westwoodii* | 7865 (4.78) |
| *Trachyaretaon carmelae* | 4744 (2.88) |
| *Xenophasmina simile* | 3448 (2.09) |


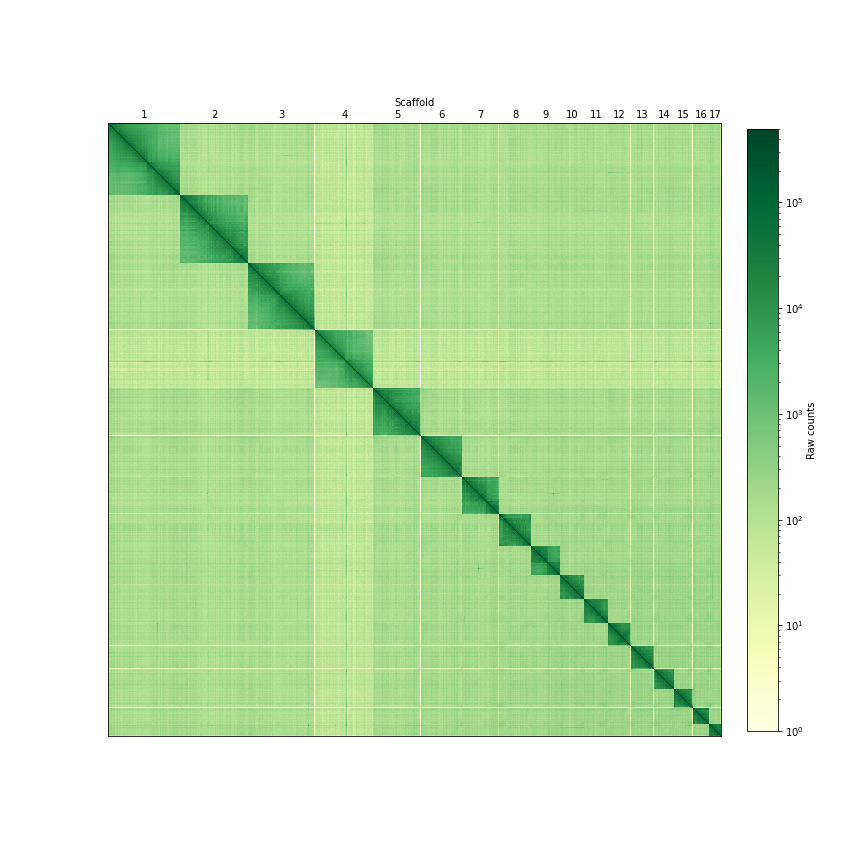


Supplementary Figure 1. Contact map of major scaffolds generated from HiRise output files using python libraries CoolTools v0.5.1 (Venev et al. 2021), Cooler v.0.8.11 (Abdennur & Mirny 2020) and MatPlotLib v3.5.3 (Hunter 2007) after mapping OmniC reads to the final scaffold assembly.


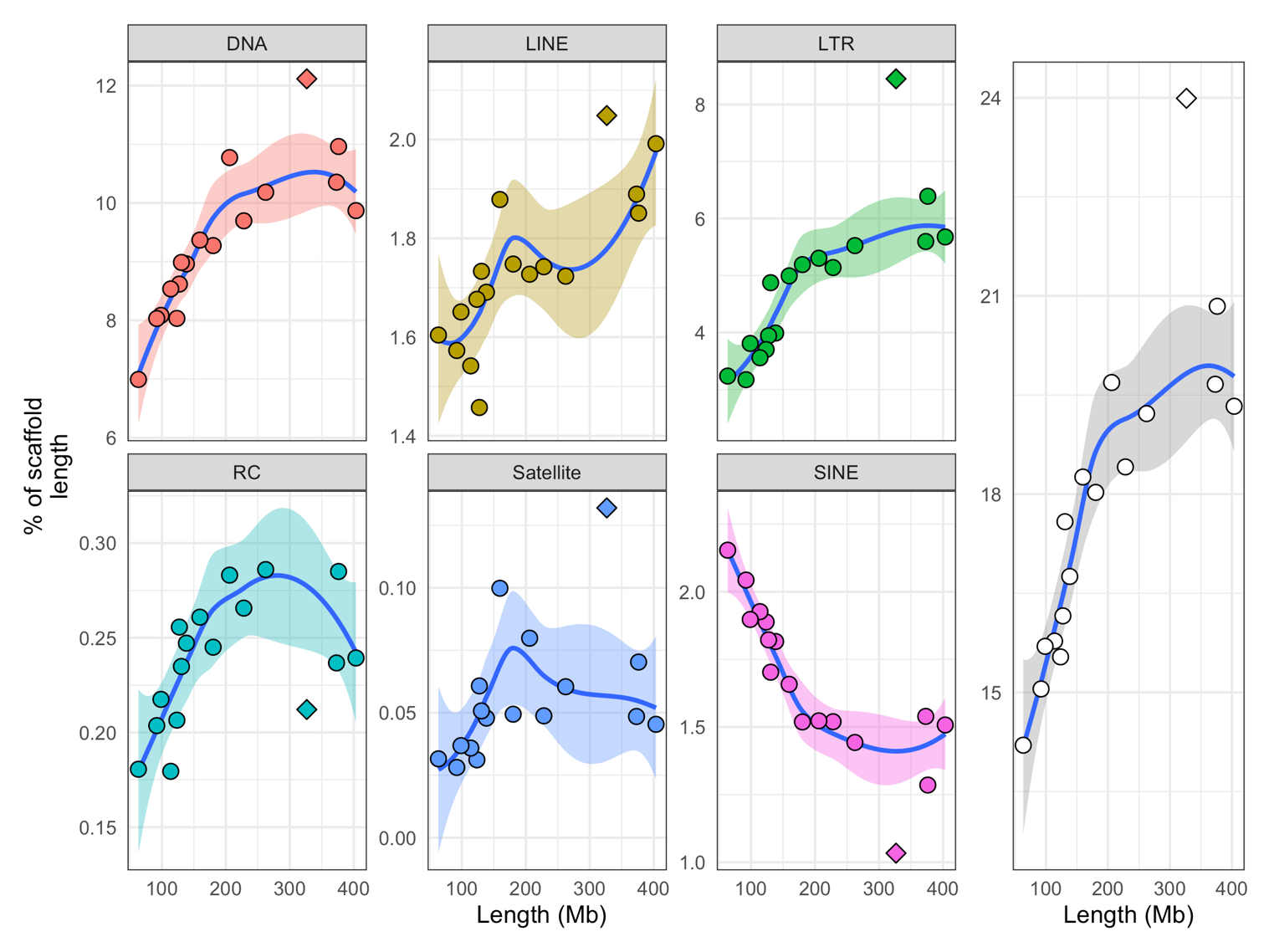


Supplementary Figure 2. Scatterplot of major scaffold length against the proportion of that scaffold covered by six classes of repetitive element. Rightmost panel shows all classes together. `Scaffold_4`, which corresponds to the X chromosome, is shown as a diamond and all autosomal scaffolds are circles. Blue lines are loess smoothed mean trendlines with shaded confidence intervals estimated with the ggplot2 functions `geom_smooth()`.


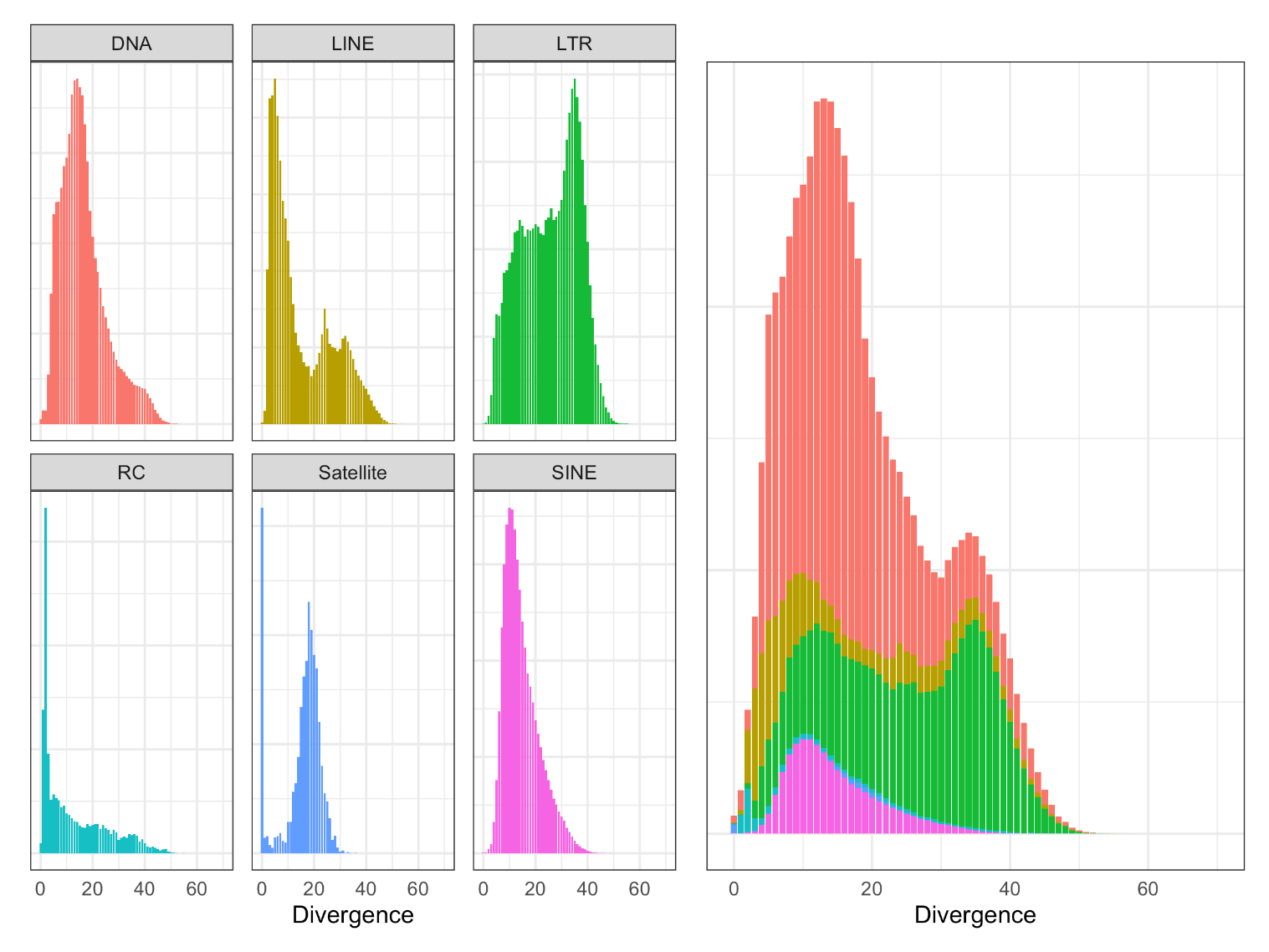


Supplementary Figure 3. Kimura 2 parameter divergence landscapes for six classes of repetitive element calculated by RepeatMasker without CpG correction.
